# Idiopathic Harlequin Syndrome: A Case Report of an Uncommon Disease

**DOI:** 10.7759/cureus.96067

**Published:** 2025-11-04

**Authors:** Mansi Singh, Lalit Kumar Tyagi

**Affiliations:** 1 General Medicine, American International Institute of Medical Sciences, Udaipur, IND; 2 General Medicine, Santosh Medical College and Hospital, Santosh Deemed to be University, Ghaziabad, IND

**Keywords:** autonomic disorder, herlequine syndrome, hyperhydrosis, rare neurological degenerative disease, unilateral facial flushing

## Abstract

Harlequin syndrome is a rare autonomic disorder presenting with unilateral facial flushing and sweating due to sympathetic dysfunction, often idiopathic but occasionally secondary to an underlying pathology.

We report a 52-year-old woman with a three-year history of left-sided hemifacial flushing and hyperhidrosis, most noticeable on exertion or heat exposure. She had comorbid diabetes, hypertension, and dyslipidemia. Neurological examination was normal. Exercise provocation reproduced the symptoms, and the iodine-starch test confirmed hyperhidrosis on the left side with anhidrosis on the right. Imaging excluded secondary causes, leading to a diagnosis of idiopathic Harlequin syndrome.

The condition results from the disruption of sympathetic vasomotor pathways at the T2-T3 level, with compensatory overactivity on the unaffected side. Although benign, it can cause cosmetic and psychosocial concerns. Management includes reassurance, botulinum toxin injections, stellate ganglion blockade, or sympathectomy. Our patient opted for botulinum toxin therapy with ongoing follow-up.

This case highlights the importance of recognizing idiopathic Harlequin syndrome and excluding secondary causes to avoid unnecessary interventions.

## Introduction

Harlequin syndrome is a rare autonomic disorder characterized by unilateral facial flushing and sweating due to sympathetic dysfunction. First described by Lance et al. in 1988 [1], it typically presents with a sharp midline demarcation between the affected and unaffected sides of the face. The condition is more prevalent in women and is often idiopathic and benign. In approximately one-sixth of cases, an underlying organic cause may be identified. When associated with other dysautonomic syndromes, such as Horner's syndrome, it is referred to as the Harlequin sign [2]. The hallmark symptom prompting medical consultation is localized anhidrosis of the hemiface, often without an identifiable cause.

While most cases are idiopathic, it may occasionally result from structural lesions along the sympathetic pathway. We report a rare case of idiopathic Harlequin syndrome presenting in a young female with isolated facial anhidrosis, emphasizing the importance of bedside autonomic testing and the role of imaging in excluding secondary causes.

## Case presentation

Presentation and clinical findings

A 52-year-old woman presented with a longstanding history of left-sided facial flushing and excessive sweating, particularly noticeable during physical exertion and in hot weather. These symptoms had been present for the past three years. The patient had a history of diabetes, hypertension and dyslipidemia for the past 10 years.

**Figure 1:**
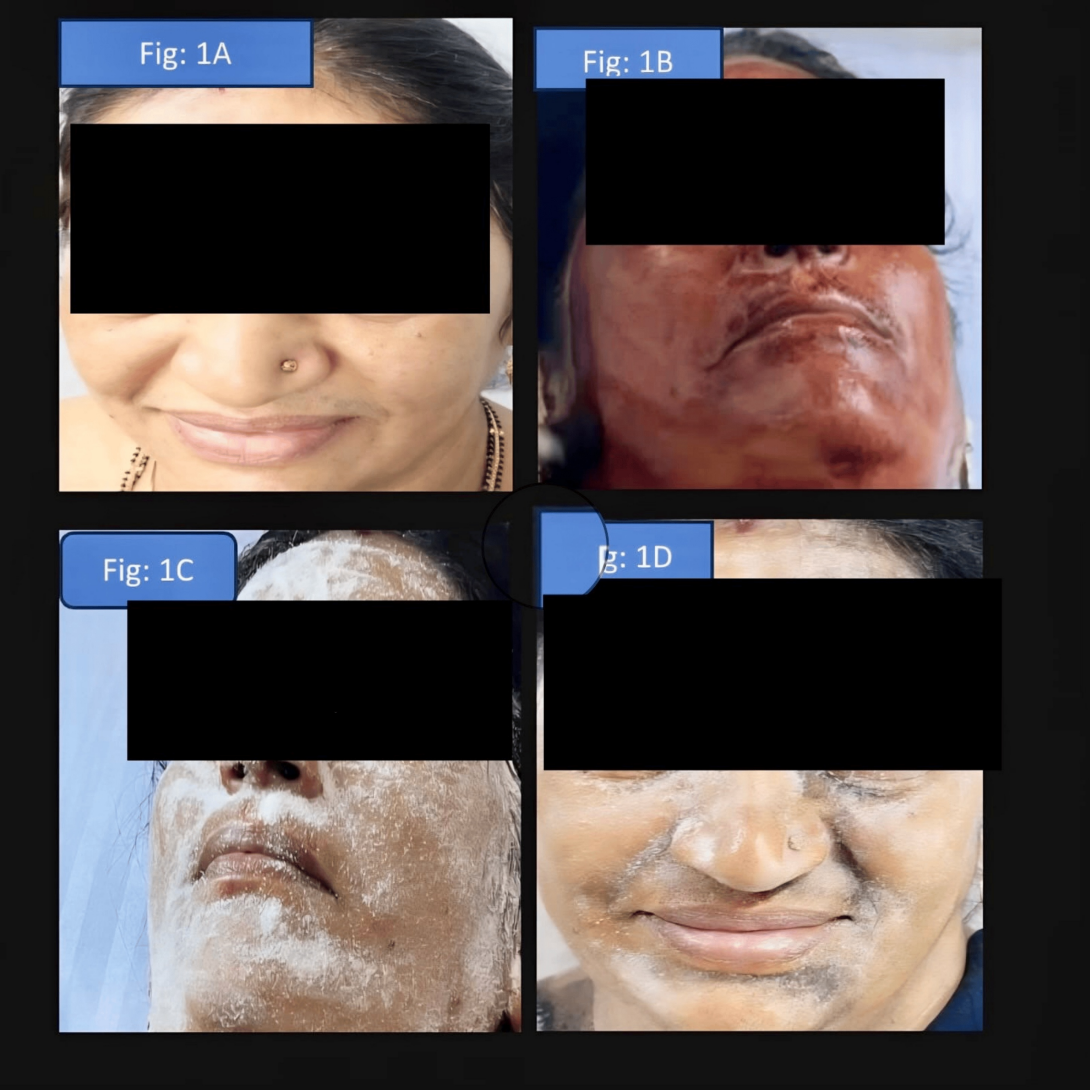
A: Baseline frontal view before testing. B: Supine view showing starch application with white powder distributed. C: Post-iodine application showing discoloration on the left side consistent with intact sweating. D: Close-up demonstrating absence of staining on the right hemiface (anhidrosis). Only one patient is discussed in this case report. The additional image included in this figure serves purely as a representative illustration to demonstrate the typical presentation of Harlequin syndrome.

On examination, the patient's dermatological assessment revealed no abnormalities or facial asymmetry. Neurological evaluation, including cranial nerve assessment, was unremarkable. To objectively assess the reported symptoms, a stress test involving 20 minutes of running was conducted. Post-exercise, the patient exhibited pronounced erythema and hyperhidrosis on the left side of the face, neck, and upper limb, especially in the suborbital region where fine telangiectasia was observed (Figure 1A). The right side remained dry and showed no erythema.

Then, iodine starch test also known as minors test was performed to evaluate sweating. To assess sudomotor function, an iodine-starch test was conducted. A thin layer of iodine solution followed by starch powder was applied symmetrically over both sides of the patient’s face (Figure 1B, C). Upon mild thermal stimulation, the normal side exhibited a bluish-black discoloration, indicating intact sweating, whereas the contralateral side showed no color change, consistent with anhidrosis (Figure 1D). This asymmetrical response corroborated the diagnosis of Harlequin syndrome, reflecting a unilateral sympathetic dysfunction affecting facial vasomotor and sudomotor activity.

Diagnosis

Based on the clinical presentation and confirmatory test by iodine starch test and exclusion of secondary causes through imaging, a diagnosis of idiopathic Harlequin syndrome was established.

## Discussion

Facial sweating and vasomotor control are mediated by sympathetic nerve fibers originating in the hypothalamus and descending through the lateral horn of the spinal cord at the T2-T3 levels [3,4]. Harlequin syndrome represents a dysfunction of this sympathetic pathway, typically involving the T2-T3 roots or their fibers, and occasionally extending to the sympathetic and parasympathetic components of the stellate ganglion. The resulting unilateral interruption of sympathetic outflow leads to ipsilateral absence of facial flushing and sweating in response to thermal or emotional stimuli [5]. In contrast, the contralateral side often exhibits compensatory sympathetic hyperactivity, causing excessive flushing and perspiration [6].

Episodes are commonly triggered by exertion or heat exposure, as observed in our patient. The distinctive half-flushed facial appearance resembles the two-toned mask of the Harlequin figure in European folklore [7]. When unilateral facial anhidrosis or flushing is identified, the underlying lesion is usually ipsilateral [8]. Reported secondary causes include cervicobrachial plexus tumors such as neuroblastoma [9], diabetic neuropathy, Guillain-Barré syndrome, Bradbury-Eggleston syndrome, toxic goiter, spinal cord infarction, and carotid artery dissection [3]. Harlequin syndrome has also been described as a rare postoperative complication following cervical spine surgery [10].

In many cases, however, the condition is idiopathic. Guillotton et al. reviewed 108 cases and found that 59 had no identifiable cause [11]. Spontaneous recovery is uncommon, and ongoing studies continue to explore its pathophysiology [4]. In our case, the absence of structural abnormalities on MRI of the brain and cervicothoracic region supported an idiopathic etiology.

Differential diagnoses considered included Horner’s and Ross syndromes, both associated with asymmetric facial sweating. Normal pupillary size and reactivity excluded Horner’s syndrome, which typically presents with miosis and ptosis due to oculosympathetic disruption. Ross syndrome was ruled out based on the absence of segmental hypohidrosis, areflexia, and other autonomic deficits. Collectively, these findings confirmed idiopathic Harlequin syndrome.

From a management perspective, idiopathic cases usually require no intervention beyond reassurance. For patients distressed by cosmetic asymmetry, botulinum toxin injections on the hyperactive side can help restore facial symmetry [12-15]. This minimally invasive approach is preferred over procedures such as sympathectomy or stellate ganglion blockade. In our case, the patient opted for periodic botulinum toxin therapy after being informed of the benign and stable nature of the condition.

## Conclusions

Harlequin syndrome, though rare, can be diagnosed clinically through careful observation of unilateral facial flushing and confirmed by simple bedside autonomic testing. Awareness of this condition is essential to differentiate it from other causes of asymmetric sweating, such as Horner’s and Ross syndromes. Recognition of its benign nature prevents unnecessary investigations and anxiety. This case highlights the value of detailed clinical examination in identifying rare autonomic disorders.

In our case, the patient is being monitored regularly, and treatment with botulinum toxin injections every six months has been initiated. This approach will allow us to evaluate both the therapeutic efficacy and cost-effectiveness of this alternative, which will be the focus of future studies. Diagnosis relies on careful neurological examination and MRI-based evaluation. Prognosis is usually benign, and management focuses on treating the underlying cause.
